# Supplementary material for: Exploring the Contribution of the AcrB Homolog MdtF to Drug Resistance and Dye Efflux in a Multidrug Resistant E. coli Isolate
Source: Antibiotics (Basel). 2021 Apr 28;10(5):503. doi: 10.3390/antibiotics10050503 (PMC8145115; doi:10.3390/antibiotics10050503)
Supplement: Supplementary file 1 [file antibiotics-10-00503-s001.zip › antibiotics-1194657-supplementary.pdf]

antibiotics

MDPI

Communication

Exploring the Contribution of the AcrB Homolog MdtF to Drug Resistance and Dye Efflux in a Multidrug Resistant *E. coli* Isolate

Sabine Schuster [1,*], Martina Vavra [1], Ludwig Greim [1] and Winfried V. Kern [1,2]

1 Division of Infectious Diseases, Department of Medicine II, University Hospital and Medical Center, 79106 Freiburg, Germany; martina.vavra@uniklinik-freiburg.de (M.V.); ludwig.greim@med.uni-duesseldorf.de (L.G.)
2 Faculty of Medicine, Albert-Ludwigs-University, 79106 Freiburg, Germany; winfried.kern@uniklinik-freiburg.de
* Correspondence: sabine.schuster@uniklinik-freiburg.de

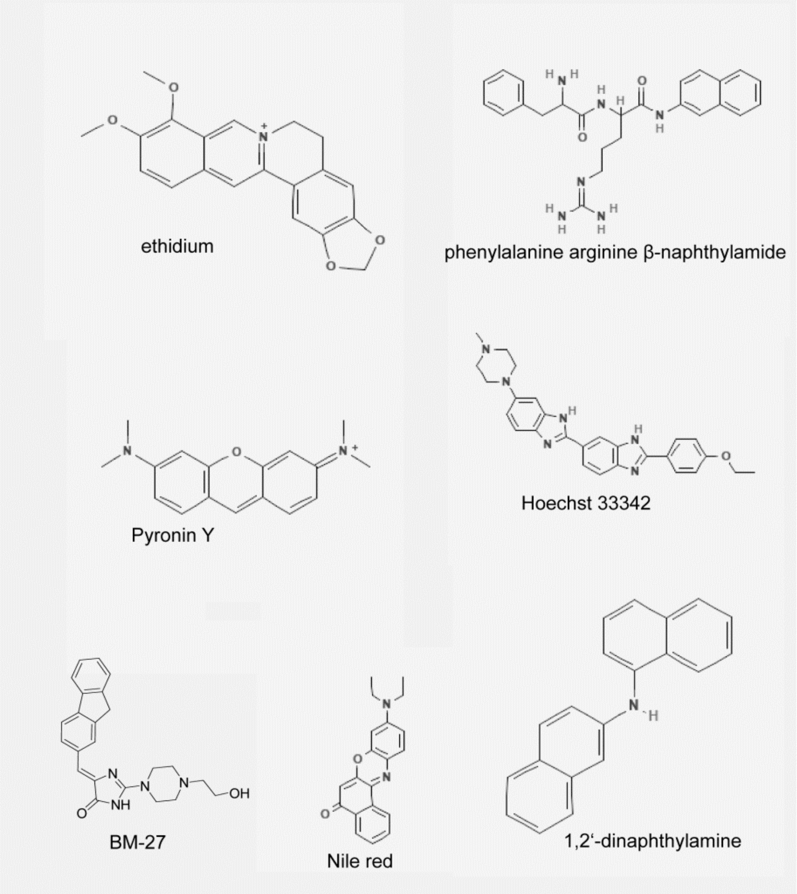

Figure 1. Dyes used in the study of the KUNΔ*acrB*/*mdtF* mutant. Phenylalanine arginine β-naphthylamide was cleaved to the fluorescent dye naphthylamine within the bacterial cell. Structures were from Pubchem (https://pubchem.ncbi.nlm.nih.gov/compound/) [1] except the structure of the piperazine arylideneimidazolone BM-27 [2] that was provided from Jadwiga Handzlik (Department of Technology and Biotechnology of Drugs, Jagiellonian University Medical College, Faculty of Pharmacy, Kraków, Poland).

Table S1. Further drug susceptibilities of the MDR *E. coli* isolate KUN9180 and derived knockout mutants.

	MIC in µg/mL [1]							
***E. coli* strain/mutant**	**LVX**	**MXV**	**GEP**	**TET**	**CHL**	**LZD**	**CLI**	**RIX**
KUN9180	32	16	2	64	4	256	64	16
KUNΔ*acrB*	4	2	0.06	16	1	16	8	4
KUNΔ*acrB*Δ*mdtF*	4	2	nd	16	1	16	8	4
KUNΔ*tolC*	4	2	0.03	16	1	16	8	4

[1] MIC, minimal inhibitory concentration (the median of ≥ 7 independent assays is shown); nd, not determined; LVX, levofloxacin; MVX, moxifloxacin; GEP, gepotidacin; TET, tetracycline; CHL, chloramphenicol; LZD, linezolid; RIX, rifaximin.

References

1. Kim, S.; Chen, J.; Cheng, T.; Gindulyte, A.; He, J.; He, S.; Li, Q.; Shoemaker, B. A.; Thiessen, P. A.; Yu, B.; Zaslavsky, L.; Zhang, J.; Bolton, E. E. PubChem in 2021: new data content and improved web interfaces. *Nucleic Acids Res* **2021**, *49*, D1388-D1395. 5957164 [pii];10.1093/nar/gkaa971 [doi].
2. Bohnert, J. A.; Schuster, S.; Kern, W. V.; Karcz, T.; Olejarz, A.; Kaczor, A.; Handzlik, J.; Kiec-Kononowicz, K. Novel piperazine arylideneimidazolones inhibit the AcrAB-TolC Pump in *Escherichia coli* and simultaneously act as fluorescent membrane probes in a combined real-time influx and efflux assay. *Antimicrob Agents Chemother* **2016**, *60*, 1974-1983. AAC.01995-15 [pii];10.1128/AAC.01995-15 [doi].
